# Unraveling the Health-Protective Effects of Sense of Coherence in Dementia Caregiving

**DOI:** 10.1093/geroni/igaa057.1143

**Published:** 2020-12-16

**Authors:** Doris Yu

**Affiliations:** The University of Hong Kong, Hong Kong, China

## Abstract

The health protective effects of sense of coherence in a stressful encounter is well recognized. Representing the extent an individual can comprehend, manage and make meaning out of a caregiving situation, little is known about how SOC shapes the dementia caregiving process in a way to benefit the carers’ health outcomes. This exploratory study recruited 401 family cares of dementia in Hong Kong. Over 30% of them reported moderate to severe depression, and those with low sense of coherence were most affected. Further analysis indicated that the health protective effects of SOC was mainly mediated through a higher level of perceived gain and more effective coping. In particular, those who can make meaning out of the caregiving situation were most benefited by such a mechanism. The study provides direction on ways to strengthen the health risk identification for family carers and enhance their role adaptation. After this presentation, the participants are able to: 1) Understand the role of sense of coherence in shaping the health of dementia family caregivers. 2) Understand the mediating pathways which explain the health benefit of sense of coherence for dementia family caregivers. □ Gain critical insights for developing health risk identification strategies and tailored interventions to protect the dementia family caregivers from depression.

